# Vaccine development: obligate intracellular bacteria new tools, old pathogens: the current state of vaccines against obligate intracellular bacteria

**DOI:** 10.3389/fcimb.2024.1282183

**Published:** 2024-03-19

**Authors:** E. J. van Schaik, A. P. Fratzke, A. E. Gregory, Jennifer E. Dumaine, J. E. Samuel

**Affiliations:** ^1^ Department of Microbial Pathogenesis and Immunology, School of Medicine, Texas A&M University, Medical Research and Education Building, Bryan, TX, United States; ^2^ Charles River Laboratories, Reno, NV, United States; ^3^ Department of Physiology and Biophysics, University of California, Irvine, Irvine, CA, United States; ^4^ Department of Veterinary Pathobiology, School of Veterinary Medicine, Texas A&M University (TAMU), College Station, TX, United States

**Keywords:** vaccines, obligate intracellular bacteria, vector-borne disease, *Coxiella*, *Anaplasmataceae*, *Rickettsiaceae*

## Abstract

Obligate intracellular bacteria have remained those for which effective vaccines are unavailable, mostly because protection does not solely rely on an antibody response. Effective antibody-based vaccines, however, have been developed against extracellular bacteria pathogens or toxins. Additionally, obligate intracellular bacteria have evolved many mechanisms to subvert the immune response, making vaccine development complex. Much of what we know about protective immunity for these pathogens has been determined using infection-resolved cases and animal models that mimic disease. These studies have laid the groundwork for antigen discovery, which, combined with recent advances in vaccinology, should allow for the development of safe and efficacious vaccines. Successful vaccines against obligate intracellular bacteria should elicit potent T cell memory responses, in addition to humoral responses. Furthermore, they ought to be designed to specifically induce strong cytotoxic CD8+ T cell responses for protective immunity. This review will describe what we know about the potentially protective immune responses to this group of bacteria. Additionally, we will argue that the novel delivery platforms used during the Sars-CoV-2 pandemic should be excellent candidates to produce protective immunity once antigens are discovered. We will then look more specifically into the vaccine development for *Rickettsiaceae*, *Coxiella burnetti*, and *Anaplasmataceae* from infancy until today. We have not included *Chlamydia trachomatis* in this review because of the many vaccine related reviews that have been written in recent years.

## Introduction

The disease burden caused by many obligate intracellular bacteria described in this review has increase at least 10-fold since the year 2000 in the USA, as reported by the CDC. Ticks transmit most of these obligate intracellular bacteria, which are responsible for >95% of all vector-borne disease in the USA (Eisen et al., 2017). The expansion of ticks’ territories over the last century has contributed to this rise in cases, which will continue because of climate change resulting in the further expansion of ticks into new regions (Sonenshine, 2018). Another obligate intracellular bacterium not known but potentially transmitted by ticks is *Coxiella burnetii* where cases of Q fever have doubled in the USA in the same period (Cherry et al., 2022). On the opposite side of the world *Orientia tsutsugamuschi* causes >1 million cases a year of scrub thyphus transmitted by chiggers, which is found primarily in southeast Asia but has an expanding territory and where the emergence of antibiotic-resistant strains is a significant concern (Luce-Fedrow et al., 2018). Recently, *O. tsutsugamuschi* was found in chiggers in North Carolina, indicating their presence in the USA, although the spread of disease has not occurred to date (Chen et al., 2023). The rise of infections and expansion of vector territories for these obligate intracellular bacteria are making them a rising public health concern. Additionally, at the early stages of disease, most of these infections present as non-specific flu-like illnesses, making diagnosis problematic. Therefore, although these infections can currently be treated effectively with doxycycline, the potential for under or misdiagnosis and the possibility for further acquired antibiotic resistance in these bacteria still makes vaccination the best strategy to mitigate disease (Strickman et al., 1995; Rolain et al., 2005; Mcclure et al., 2017). It is our view that any vaccination strategy that stimulates a Th1 mediated response with the production of pathogen specific antibodies, CD4+ T cell, and CD8+ cytotoxic T cell will be most effective for any of the obligate intracellular bacteria discussed in this review. Several strategies including novel antigen/adjuvant mixtures or delivery platforms could accomplish this goal.

Vaccination is one of the most important medical advances and has nearly eradicated several diseases responsible for significant morbidity and mortality in both humans and many animal species. A recent study estimated the number of potential human deaths eliminated by vaccination as 50 million in just the new millennium (2000-2019) (Toor et al., 2021). However, none of the approved vaccines for human or animal use are for the obligate intracellular bacteria discussed in this review. All these zoonotic diseases would benefit from a one health initiative. And although there is a critical need for animal vaccines for obligate bacteria in these genera discussed in this review, the focus will be on human vaccines. One of the main reasons there are no approved vaccines for this group of bacteria is their intracellular lifestyle because although antibodies can provide some protection, once intracellular a cell-mediated response will be necessary for clearance. Furthermore, since most of these bacteria actively subvert the immune system a vaccine strategy that produces a robust humoral and cell-mediated immune response will have a better chance of providing protection. Challenges at the clinical trial stage will include defining novel cell-mediated correlates of protection a challenge for any vaccine that relies on humoral and cell-mediated immunity. Additionally, humoral correlates of protection will be more difficult to establish as neutralization is often not mechanisms of protection. This review aims to provide a comprehensive description about what is known about the immune responses to natural infection and how the information can guide human vaccine development. Additionally, vaccine immunology necessary for understanding the components of protection will be discussed, followed by several new vaccinology approaches that could aid the development of successful vaccines for these pathogens.

## Vaccination immunology specific for obligate intracellular bacteria

**Figure 1:**
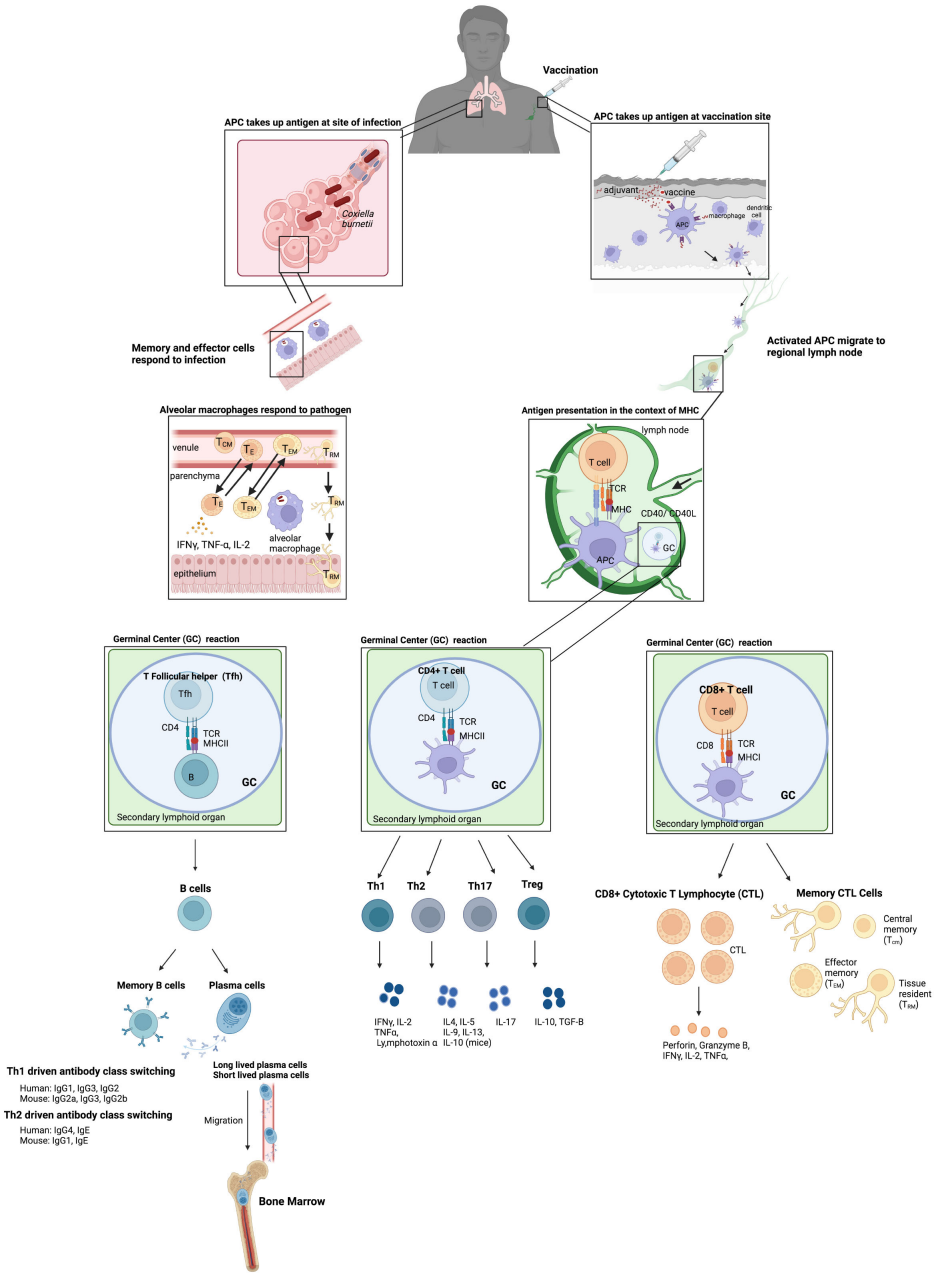
Basic vaccine immunology. Vaccination usually occurs via injection intramuscularly in humans where antigens are taken up by APCs at the site and are also activated by adjuvant PAMPs included in the vaccine which stimulate the innate immune system. These activated APCs then migrate to the regional lymph nodes to present these antigens to naïve T cells in the context of MHCI and MHCII to T cell receptors (TCR). CD4+ T cells and CD8+ T cells then develop into effector and memory populations. The memory population of T cells is separated into 3 different types: central memory (T_CM_, effector memory (T_EM_), and tissue resident (T_RM_). The CD4+ T helper cells also drive B cell development in germinal centers by providing help after B cell receptors recognize soluble antigen found in the lymph nodes. B cells then undergo class switching and develop into antibody secreting plasma cells and memory cells. This results in long-term immunity to the vaccine material. These memory cells can then be maintained at several sites including the bone marrow for long lived plasma cells (LLPCs) which can secrete antibodies long term. In addition, infection after vaccination results in release of cytokines at the site of infection by T_RM_ and other cells of the innate immune system which can then recruit other memory T cells to the site of infection. The example of an infection after vaccination shows *C. burnetii* in the lungs, particularly in the alveoli where alveolar macrophages uptake *C. burnetii* and key players in the Th1 type response produced after vaccination which provides protection.

Most vaccines are injected with needles, where antigens are taken up by antigen presenting cells (APCs) at the site of vaccination which can also be activated by adjuvant-type pathogen-associated molecular patterns (PAMPs) (**Figure 1**). These activated APCs migrate to regional lymph nodes to present antigens to naïve T cells in the context of MHCI and MHCII to T cell receptors (TCR), which are assumed to be essential for cellular immunity to obligate intracellular bacteria (**Figure 1**). CD4+ T cells are more important for clearance of vacuolar bacteria (*C. burnetti*, and *Anaplasmataceae*), whereas CD8+ T cells are important for the clearance of cytoplasmic *Rickettsiaceae*. However, it is the view of the authors that any vaccine formulation that can stimulate both pathogen specific CD4+ and CD8+ T cells should provide more protection and either alone. The importance of various immune responses will be discussed in the pathogen specific sections. CD4+ and CD8+ T cells then mature into effector and memory populations. The memory population of T cells can be separated into 3 different types: central memory (T_CM_), effector memory (T_EM_), and tissue resident (T_RM_) (**Figure 1**). Effective vaccines for obligate intracellular bacteria likely require a strong T_RM_ response, particularly since T_RMS_ are found in the skin, lungs, liver, and intestines. T_RMS_ rapidly respond to local antigen causing cytokine release and recruitment of circulating memory T cells (**Figure 1**). A strong T_RM_ response after mucosal immunization with inactivated *C. trachomatis* has been observed, which demonstrated that in mice optimal clearance is dependent on T_RMS_ and circulating memory T cells supporting our view that T_RMS_ will be important for vaccine induced protection against the other obligate intracellular bacteria discussed in this review (Stary et al., 2015). CD4+ T helper cells also drive B cell development by providing help after B cell receptors recognize soluble antigen found in the lymph nodes. This is provided by a subset of CD4+ T helper cells called T follicular helper cells (Tfh) which allow B cells then undergo class switching and develop into antibody secreting plasma cells, long-lived plasma cells (LLPCs), and memory cells (**Figure 1**) (Olatunde et al., 2021). LLPCs reside in bone marrow and can secrete antibodies for years, decades, or longer in humans. On the other hand, short-lived plasma cells are usually found in extrafollicular locations (Bortnick and Allman, 2013). It is now recognized that LLPCs provide long-term and sustained production of antibodies, and therefore any effective vaccine to an obligate intracellular that requires an antibody response should stimulate the production of LLPCs (Lightman et al., 2019). Passive transfer of sera to naïve mice provides protection against many obligate intracellular bacteria including *Ehrlichia chaffenesis* and *Rickettsia conorii* (Feng et al., 2004; Yager et al., 2005). However, passive transfer of sera to athymic mice was not protective for several obligate intracellular bacteria including *Coxiellla burnetii* and *R. akari* suggesting that T cells are involved in antibody mediated protection in some cases (Kenyon and Pedersen, 1980; Zhang et al., 2007) Therefore, although antibodies are important components of the protective immune response to obligate intracellular bacteria, a cell-mediated response should also be stimulated to confer the most efficacious level of protection ensuring that both branches of the immune system are involved in the protective response.

A cell-mediated immune response with CD4+ and/or CD8+ T cells providing most of the protection is generally required to control disease caused by obligate intracellular bacteria. Therefore, an effective immune response that requires CD4+ T cells should skew towards a Th1 phenotype with CD4+ T cells that secrete IFNγ for clearance of obligate intracellular bacteria, in addition, Th1 responses stimulate cytotoxic CD8+ T cells through the secretion of IFNγ, and IL-12 (Turner et al., 2021). A Th1 skew is also partially dependent on Th17 cells, which are beneficial to protective immune responses to obligate intracellular bacteria including *C. trachomatis* by contributing to Th1 modulation, neutrophil recruitment, and dendritic cell (DC) regulation (Li et al., 2018; Nguyen et al., 2021). DCs are the APCs that drive this type of response and determine what type of CD4+ T helper cells develop. CD4+ T helper cells come in a range of unique forms including Th1, Th2, Th17, Treg, and Tfh to name a few (**Figure 1**). The cytokines present during T cell expansion, after binding of the TCR to peptide bound in MHCII, determine the types of T helper cells that result. Conventional DCs (cDCs) are the most abundant DCs and are found in all tissues, therefore, these are the APCs that ingest antigen at the site of vaccination. Induction of Th1 cells occurs after cDC uptake of antigen with stimulation of TLR3, TLR9, or TLR11/12 and self-stimulation with IFNγ (Hilligan and Ronchese, 2020). Adjuvants that stimulate those TLRs are logical adjuvants to include in vaccines against obligate intracellular bacteria. This type of stimulation causes the cDC to release IL-12 and IL-27, both necessary to skew CD4+ T helper cells into Th1. In addition, vaccination that induces the development of CD4+ cytotoxic T lymphocytes (CTLs), which are induced after chronic viral infections with HIV-1 and described as CD4+CD28-Perforin+ T cells, should be beneficial against intracellular bacterial infections (Appay et al., 2002). These CTL CD4+ T cells are induced after a Th1 response. Th1 cells are also important for enhancing CD8+ T cell-mediated immunity by secreting IFNγ, TNFα, IL-2, IL-3, and GM-CSF (Zander et al., 2019). In addition to shaping the CD4+ T helper cell response, cDCs are also the most effective at cross-presentation of antigens on MHCI through the vacuolar and endosome-to-cytosol pathways and are also required for CD8+ cytotoxic T cell response (Embgenbroich and Burgdorf, 2018). Therefore, any delivery system that targets antigens to cDCs could be valuable in a vaccine to any obligate intracellular bacterium discussed in this review since targeting a Th1/Th17 with a strong cytotoxic T cell response should be required for protection.

Vaccine formulations will likely have to include both T cell and B cell antigens to confer protection against obligate intracellular bacteria highlighting that both cell-mediated and humoral responses are optimal to clear obligate intracellular bacteria. The concept that a humoral plus CD4+ T and CD8+ T cells response confers optimal protection is exemplified by a vaccine developed against *Mycobacterium tuberculosis*. Engineering the BCG vaccine strain to produce listeriolysin O and deleting Urease C created VMP1002. This live attenuated vaccine strain was still able to confer a humoral and Th1 CD4+ T cell response like BCG, but additionally stimulates CD8+ cytotoxic T cells and afforded better protection than BCG in animal models (Grode et al., 2005). This vaccine is now in phII/phIII clinical trials in India. We believe any vaccine strategies that stimulate a strong humoral plus CD4+ Th1 response and additionally stimulate a CD8+ cytotoxic response will be the most efficacious (Nieuwenhuizen et al., 2017). Therefore, any vaccine developed should induce humoral and cell-mediated responses to provide the best protection.

## Correlates of protection that should be developed for obligate intracellular bacterial vaccines

Defining the CoPs is an important step in licensing an efficacious vaccine. Understanding which branch(es) of the immune system are essential in a protective response determines what are the CoPs. If CoPs are well defined, it is simpler to develop efficacious vaccines then if empirical studies are required for each new antigen. For example, serum antibody concentrations of 0.15 to 1.0 mg/mL to the polysaccharide of *H. influenzae* type b (HiB) are a CoP that can be used to develop new vaccines against HiB polysaccharide (Granoff and Lucas, 1995). More specifically this is a “mechanistic CoP” (mCoP), meaning it is a specific immune function that confers protection. Antibodies still represent the primary correlate of immunity for most vaccines that are licensed (Plotkin, 2013). In contrast, a non-mechanistic CoP (nCoP) may be correlated to protection or contributes to protection but is not a direct measurement of an immune response (Plotkin and Gilbert, 2012). Infection resolved cases can be used as a starting point to define CoPs for obligate intracellular bacteria, that is if infection resolved cases provide decent protection, which is not the situation of all the pathogens in this review. In addition, the ability to subvert the immune system creates issues because even if infection resolved cases provide protection this may not be the best protection because of inherent subversion mechanisms. CoPs for obligate intracellular bacterial vaccines will need to be defined for licensing and will rely not only on humoral responses which are well defined CoPs but also on cell-mediated responses and to date there are no approved vaccines with only cell-mediated CoPs.

An appropriate animal model that mimics human disease is essential to determining CoPs. Vaccination in an appropriate animal model can be used to evaluate CoPs through a variety of experimental techniques including depletion studies, antibody titers, and cellular assays. This is often challenging for obligate intracellular bacteria, especially those that are transmitted through vectors; however, advancements have been made and will be highlighted in pathogen specific sections. Although antibodies can have several functions, one of the most common CoPs are neutralizing antibodies. Neutralization implies coating the virus, bacteria, or toxin with antibodies and preventing them from binding to host cells. This is the CoP for of all the vaccines approved for preventing Sars-Cov-2 infection for the recent pandemic (Carrillo et al., 2021). Other antibody mediated mechanisms include opsonization and activation of the complement cascade. The mechanisms of antibody mediated immunity to obligate intracellular bacteria are often difficult to ascertain because many of these pathogens grow within immune cells even after binding antibodies (Shannon et al., 2009). Defining appropriate humoral mediated correlates of protection will be essential for licensing of any obligate bacterial vaccine that depends on this type of immunity. Cell-mediated CoPs are not as well defined, which has made the development of viral and obligate intracellular bacterial vaccines that will rely on cell-mediated protection difficult to evaluate (Barker et al., 2009).

To date there is no licensed vaccine that produces only T cell protection on the market. However, induction of CD4+ T cells has been used as a CoP for Shingrix the vaccine licensed for use against shingles in adults over 50 (Ogunjimi and Beutels, 2018). The gold standard for testing antigen specific T cell responses after vaccination is an ELIspot usually for IFNγ which can be used in mice, non-human primates (NHPs), and humans during vaccine development (Flaxman and Ewer, 2018). The major disadvantage of ELIspots is that there is no information about the phenotypes of the cells secreting IFNγ. This can be circumvented by using flow cytometry to separate cells into CD4+ and CD8+ populations that express IL-2, IFNγ, IL-4, IL-17, perforin, and granzyme B (Flaxman and Ewer, 2018). Flow cytometry can also be used to define memory populations (Flaxman and Ewer, 2018). Given that 40 different markers can now be measured using mass cytometry (CyTOF), the use of flow cytometry to identify CoPs during vaccine development will only increase and is the obvious choice for development of cellular CoPs for obligate intracellular bacteria. The above CoPs for humoral and cell-mediated responses should be used to develop safe and efficacious vaccines for all the obligate intracellular pathogens.

## Antigen selection

How do you pick antigens to be included in a vaccine? One of the earliest vaccine approaches to this problem was in fact to not pick at all and to inactivate the entire bacterium creating a whole-cell vaccine (WCV). A bacterial pathogen is cultivated in large quantities and then inactivated by one of several methods including but not limited to formalin or another chemical inactivation, heat, or irradiation (Khan et al., 2022). The advantage of these types of vaccines is that they are highly immunogenic and usually do not require adjuvants or boosts. Surprisingly, this is effective for several obligate intracellular bacteria including *C. burnetii* (Q-VAX^®^), however, Q-VAX^®^ produces significant reactogenic responses in previously sensitized individuals and, therefore, was never licensed for use in the USA (Fratzke et al., 2022). The reactogenicity of WCVs can be reduced or eliminated and appropriate effector memory stimulated using specific antigens and epitopes alone or in solubilized cellular antigen extracts, as was demonstrated recently demonstrated in preclinical animal models for *C. burnetii* (Gregory et al., 2021). Identification of antigens can be accomplished by many approaches. Early strategies to identify dominant and subdominant antigens described as the immunome have been employed for many obligate intracellular bacteria using antiserum from previously infected individuals to identify protein antigens (Beare et al., 2008; Luo et al., 2021). However, there are inherent limitations in these approaches for the obligate intracellular bacteria, where immunogenic proteins are not necessarily protective and where antibody responses alone usually do not afford complete protection (Zhang et al., 2007). Surface exposed proteins, or sugar moieties including lipopolysaccharide (LPS) are good antigenic targets for humoral responses to the obligate intracellular bacteria that produce LPS, which excludes *Anaplasma* spp., *Ehrlichia* spp. and *O. tsutsugamuschi*, however, surface exposed protein antigens are still likely candidates for these bacteria (Luo et al., 2020). For example, the LPS from *C. burnetii* has been demonstrated to confer protection in both mice and guinea pigs (Zhang et al., 2007; Jan et al., 2023).

The advance of high-throughput sequencing gave way to the era of reverse vaccinology (Rappuoli, 2000). Reverse vaccinology has been used to identify such antigens as those used in the MenB vaccine against *Neisseria meningitidis* which represents a milestone in new vaccinology approaches (Masignani et al., 2019). Reverse vaccinology has been used extensively to identify antigens from obligate intracellular bacteria and has identified components of secretion systems and their secreted effector proteins as novel antigens (Khan et al., 2023; Sabzi et al., 2023). We are now in the era of rational vaccine design, which builds on reverse vaccinology with the addition of machine learning algorithms to identify antibody and T cell epitopes. The SARS-CoV-2 pandemic significantly advanced these approaches by providing rich data to employ novel machine-learning techniques to predict B and T cell epitopes using a single framework like DeepVacPred to the forefront (Yang et al., 2021). We, and others, predict this could accelerate the development and testing of novel vaccines for obligate intracellular bacteria. Mapping of epitopes for B and T cells, commonly referred to as immunoinformatics, is the basis for the design of epitope-based vaccines (Parvizpour et al., 2020). This approach does not require an entire antigenic protein and can remove immunodominant areas of antigens that do not confer protection.

More specifically, immunopeptidomics, which refers to the investigation and dynamics of all peptides presented by major histocompatibility complex (MHC) class I and class II using mass spectrometry will also add strategies for vaccines against obligate intracellular pathogens (Kovalchik et al., 2020). This technique has been applied recently in the development of a tuberculosis vaccine wherein 43 MHCI and 94 MCHII epitopes were identified in *M. tuberculosis* infected cells (Mayer and Impens, 2021). As presented in each of the following pathogen-specific sections, these advances have revolutionized the development of vaccines for obligate intracellular bacteria resulting in several safe and efficacious vaccine formulations ready for human-phased trials. Notwithstanding, pathogen adaption that results in reduced immunogenicity may remain an issue that requires combining the existing tools or developing new technology to overcome.

## Adjuvants

Adjuvants are vaccine components that stimulate the innate immune system to enhance the magnitude, extent, and longevity of the adaptive response to the antigens. As mentioned above, these are required additives for the new era of antigen and epitope-based vaccines. Adjuvants come in 3 basic formats: 1. as a depot that recruits antigen-presenting cells (APCs), 2. as a delivery system that helps antigen uptake by APCs, or 3. stimulate innate immune responses through pattern recognition receptors (PRRs). Once the PRRs of the innate immune system are stimulated in cDCs an adaptive immune response can result in the maturation of number of CD4+ and CD8+ T cell subsets in addition to B cells (**Figure 1**).

Alum, an insoluble aluminum salt, was the first adjuvant licensed for use in the 1920s and, until very recently, the only adjuvant licensed for use in humans. Surprisingly, the exact mechanism of Alum remains poorly understood despite decades of licensure and use. Unlike most of the new adjuvants that activate one or more PRR, it has long been assumed that Alum causes a depot effect. However, it is clear that the mechanism of action of Alum is more complex and involves enhancing the delivery of APCs, production of IL-1β, and induction of cell death releasing DNA and other damage-associated molecular patterns (DAMPs), that act as endogenous adjuvants (Del Giudice et al., 2018). There are currently 5 other adjuvants licensed for use in humans including MF59 which is included in the seasonal flu vaccine for older adults Fluad (licensed in Europe), AS01 in the shingles vaccine Singrix, AS03 in the pandemic influenza vaccine Pandemrix, AS04 in the HPV vaccine, and finally CpG in the HBV vaccine (Pulendran et al., 2021). In addition to these adjuvants, many other adjuvants are in the pre-clinical stages of development to ensure their safety and tolerance.

Overall, the goal of an adjuvant inclusion in a vaccine for most obligate intracellular bacteria is to elicit a strong Th1 cellular response resulting in antigen specific antibodies, CD4+ and/or CD8+ T cells. A strong cytotoxic CD8+ T cell response to antigens may be ideal but requires cross-presentation for subunit vaccines. This response requires the presentation of peptides in MHCI restricted fashion on APCs, usually cDCs, with proper cytokines and co-stimulation to induce T cell differentiation (Zhang and Bevan, 2011). Classically this requires peptides loaded from the cytosol into the ER and then MHCI. However, this process can occur directly from peptides in a phagosome or by transfer of peptides into the cytosol. Both processes are considered cross-presentation, which is inefficient with subunit vaccines and does not result in long-term memory CD8+ T cells. Numerous adjuvants are now under development as carriers that enhance the cross-presentation of antigens a formulation that will likely be necessary to develop safe and efficacious subunit and/or epitope vaccines for the obligate intracellular pathogens discussed in this review (Embgenbroich and Burgdorf, 2018).

## Delivery platforms

Vaccine delivery platforms comprise two different branches. One branch tests novel delivery platforms including edible vaccines, microneedle patches, or intradermal vaccines. The alternative is the basic formulation of the vaccine material and the focus of this section. For example, the SARS-CoV-2 pandemic saw the licensing of the first 2 mRNA vaccines for human use in the USA produced by Moderna, Inc. and BioNTech SE/Pfizer Inc (Barbier et al., 2022). The basic principle of an mRNA vaccine is the delivery of mRNA into the cell by a lipid nanoparticle resulting in the production of a protein in the cytoplasm that can be presented by MHCI to stimulate a cytotoxic CD8+ T cell response, in addition to stimulating CD4+ T cells and humoral responses (Verbeke et al., 2022). Furthermore, the lipid nanoparticle and the foreign mRNA can stimulate several innate immune-signaling cascades acting as adjuvants (Li et al., 2022). The ability to stimulate a variety of innate immune pathways followed by stimulation of antigen specific humoral, CD4+ and CD8+ T cell responses in addition to the ease of production should make mRNA vaccines ideal for vaccines developed against obligate intracellular bacteria once novel techniques to discovery antigens are used (Kowalzik et al., 2021). A DNA vaccine delivery method was also licensed during the SARS-Cov-2 pandemic using a non-replicating chimpanzee-Ad (adenoviral vector) in the licensed Oxford-AstraZeneca vaccine (Van Doremalen et al., 2020). DNA vaccines work with the same basic principle as mRNA vaccines, however, the DNA must first be transcribed in the nucleus and then translated into protein in the cytoplasm. This again allows the loading of MHCI to stimulate a cytotoxic CD8+ T cell response in addition to an antibody response. The only caveat with adenoviral vector vaccines is the potential for previous exposure and pre-existing immunity to the non-replicating viral vector which may limit the utility of these platforms as was seen with HIV-ad5 vaccines where having anti-ad5 antibodies actually increased the acquisition of HIV (Buchbinder et al., 2008; Mcelrath et al., 2008).

A novel delivery platform uses outer membrane vesicles (OMVs) which are produced by all Gram-negative bacteria. This technology not only has value for creating innovative vaccines against bacteria pathogens but is also being developed as a delivery platform for cancer vaccines (Zhu et al., 2021). OMVs work as a delivery platform because they can package antigens and also act directly as an adjuvant as they are composed of LPS a TLR4 agonist in addition to several other PAMPs that can stimulate TLR2 or TLR5 on the cell surface and caspase-11 and NODs in the cytoplasm (Vanaja et al., 2016). Antigens can be expressed on surrogate bacteria to produce OMVs, which is a strategy that could be applied to obligate intracellular bacteria. More specifically, genetic fusions to the C-terminus of ClyA, a pore-forming toxin, can be used to create recombinant OMVs for vaccination expressing the antigen of interest on the outside of the OMVs (Rappazzo et al., 2016). Interestingly, this strategy produces a Th1/Th17 response in vaccinated mice and was shown to confer protection after vaccination with M2e a potential universal influenza A antigen in a mouse model of influenza, the type of response would also be beneficial for vaccine development to obligate intracellular bacteria (Kim et al., 2013; Rappazzo et al., 2016). As delivery platforms continue to be developed, the ability to create safe and efficacious vaccines to obligate intracellular bacteria also advances.

## Vaccine strategies for intracellular pathogens

Except for the early WCVs, obligate intracellular bacterial pathogens have thwarted efforts made toward efficacious vaccines. Although most of these obligate intracellular bacteria can be culture *in vitro* the inability to effectively culture axenically like *C. burnetii* has significantly slowed the progress towards the development of safe and efficacious vaccines (Omsland et al., 2009; Mcclure et al., 2017). This is mainly because genetics and therefore the determination of virulence factors as lagged. It is only the last few years that novel antigen identification platforms have been used for any of the obligate intracellular pathogens. The novel methodologies and machine learning algorithms developed during the Sars-Cov-2 pandemic have made the next phases of antigen discovery imminent. Here we present the current understanding of the immunobiology of key obligate intracellular bacteria including *Rickettsiaceae*, *C. burnetti*, and *Anaplasmataceae* and the current state of vaccine development efforts to this unique group of pathogens. All of the CoPs sections for these bacteria have been determined using animal models or human infections from natural infections and although they have provided groundbreaking information the CoPs for licensing will still need to be defined in the future.

## Rickettsiaceae

### Bacteria and disease overview

**Figure 2:**
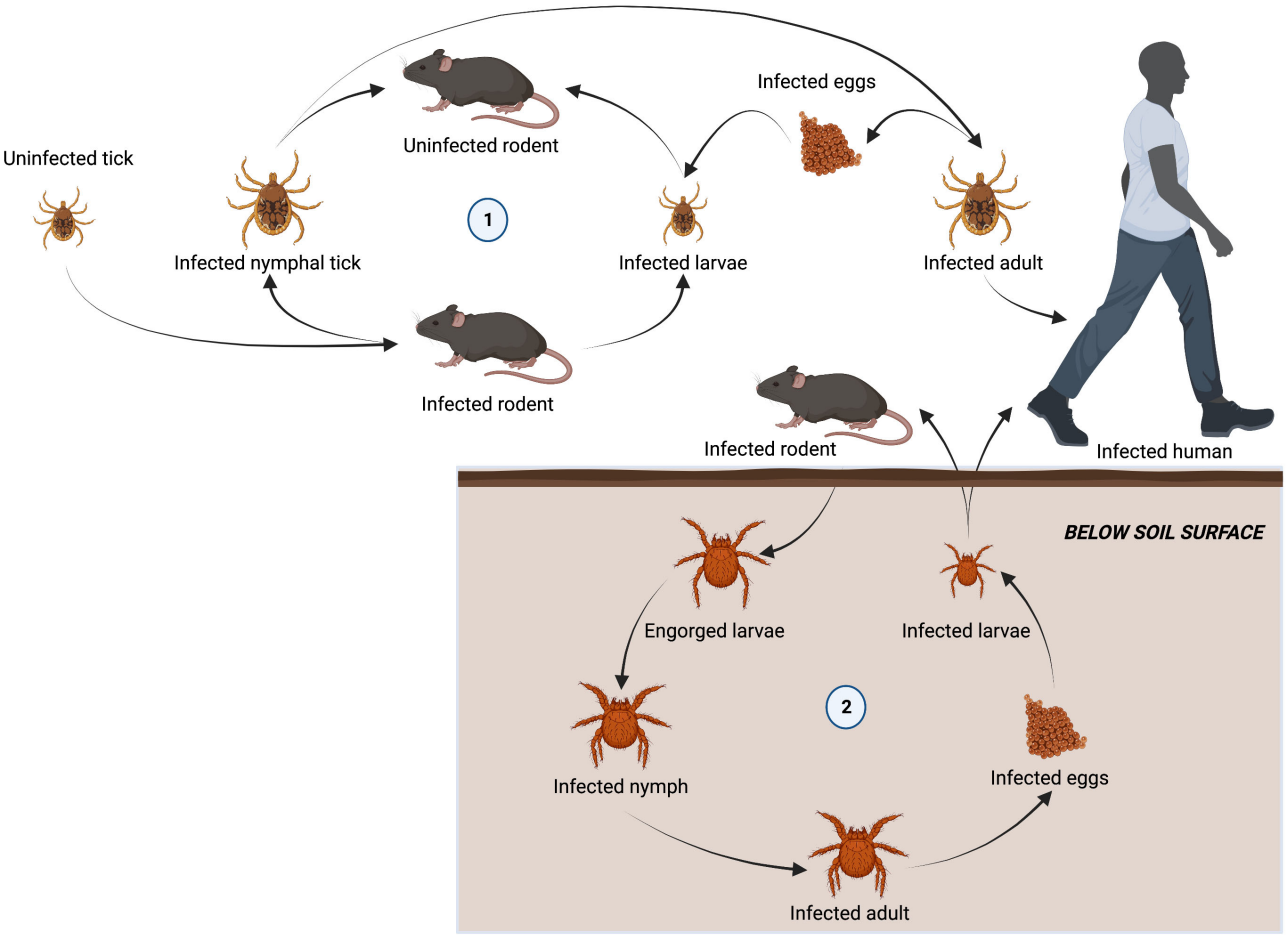
Lifecycle of arthropod vectors responsible for transmitting tick-borne diseases to humans. Ixodidae are a family of hard ticks responsible for transmitting ehrlichiosis, anaplasmosis, and rickettsioses in a lifecycle involving three-hosts. Adult females detach from a host after feeding to lay eggs. Six-legged larvae hatch from the eggs and seek out a host (often a rodent) to attach and feed. Engorged larvae leave their host and molt into nymphs before seeking out a second host (often a rodent). Nymphs drop from their host and molt into adults before attaching to a third host (often larger mammals, including humans). Adults will feed and mate on the third host before detaching and laying eggs to restart the cycle (1). Chiggers, responsible for spreading scrub typhus, spend most of their life underground. Larvae emerging from infected eggs, start to display host-seeking behavior within a few days by forming clusters on leaves and grass above the soil surface. The larvae feed on a vertebrate host (including humans), becoming engorged and increasing in size by several fold. They then detach and return to a suitable habitat on the soil surface. Over a 2-week period engorged larvae develop into a quiescent 8-legged nymphal phase. A month later, the adult finally emerges and may survive for 15 months or more. When an infected adult lays eggs, *O. tsutsugamushi* is transovarially transmitted to the offspring, maintaining their infectivity over long periods of time and restarting the life cycle.

The family *Rickettsiaceae* consists of obligate intracellular bacteria belonging to the genera *Orientia* and *Rickettsia*. These bacteria have a wide range of potential hosts, including humans, which are infected via arthropod vectors (**Figure 2**).

The genus *Orientia* (previously genus *Rickettsia)* was long considered to include only a single species, *O. tsutsugamushi*. *O. tsutsugamushi*, the causative agent of scrub typhus, is an emerging vector-born pathogen transmitted to humans during a blood meal from infected trombiculid mites (chiggers). The geographical distribution of scrub typhus was historically considered to be limited to Asia, Australia, and islands in the Pacific Ocean an area referred to as the Tsutsugamushi triangle (Kelly et al., 2009; Elliott et al., 2019). However, recent cases of scrub typhus like illness outside the Tsutsugamushi triangle coupled with efforts to broaden molecular surveillance of scrub typhus have revealed evidence of *Orientia* species far outside of the triangle region including cases in Africa, Europe, and South America and resulted the identification of at least two genetically distinct *Orientia* agents, *Candidatus* O. chuto and *Candidatus* O. chiloensis (Jiang and Richards, 2018). Given the potential severity of scrub typhus infections, a mortality rate of up to 35% if not treated early, and rising concerns over potential outbreaks of scrub typhus in vulnerable regions, efforts to develop effective vaccines have grown in recent years (Chaudhry et al., 2019).

*Rickettsiae* are a diverse genus of bacteria that can be subdivided into four main groups based on molecular, phylogenetic, and serological profiling. The first two groups are the typhus group (TG) and spotted fever group (SFG), which represent the majority of severe human-acquired rickettsiosis. The TG includes two closely related species: *R. typhi* and *R. prowazekii*, the causative agents of murine typhus and epidemic typhus, respectively. The SFG is more heterogeneous and contains most *Rickettsia species*, approximately 20, including *R. rickettsii* and *R. conorii*, the causative agents of Rocky Mountain Spotted Fever (RMSF) and Mediterranean Spotted Fever, respectively. *R. akari* and *R. felis* belong to the transitional group (TRG), so-called because they consist of features represented by SFG and TG. Finally, there is a non-pathogenic ancestral group (AG) consisting of *R. belii* and *R. canadensis*. While SFG rickettsioses are transmitted to mammals predominantly via ticks, TG and TRG rickettsiae have a much broader arthropod reservoir including fleas, mites, lice, and ticks (Osterloh, 2017). The geographical range for some of the tick species responsible for transmission of *Rickettsia* has increased in recent years, likely as a result of warmer climates (Karbowiak, 2014). This in turn has correlated with an increased incidence of rickettsioses not only in endemic regions but also where rickettsioses were rarely documented (Adem, 2019).

### CoPs

During the early stages of infection, rickettsiae invade professional phagocytes in the dermis before spreading to endothelial cells. Here, the bacteria replicate in the cytosol before initiating cell-to-cell spread either by fusing with the host membrane and budding off (*Orientia* spp.), utilizing actin-based motility (SFG), or rupturing the parasitized host cell (TG). Due to the obligate intracellular lifecycle and innate evasion strategies of rickettsiae, seroconversion following infection is significantly delayed (Fournier et al., 2002). Diagnostic antibody titers in *R. typhi* and *R. conorii* infected patients do not often develop until around two to three weeks after an initial infection (Dumler et al., 1991; Fournier et al., 2002). Additionally, a study using inactived *R. rickettsii* lead to a rise in humoral response in a subset of vaccinees but did not prevent disease (Dupont et al., 1973). Thus, humoral immunity plays only a minor role in mediating host protection of primary infection but may play a more significant role in limiting secondary infections (Chan et al., 2010). During the early stages of a primary infection, antibodies produced in the absence of CD4+ T cell help are less protective than antibodies generated with CD4+ T help. In a study involving convalescent serum from athymic nude mice donors, passive transfer of antibodies was able to confer protection in a euthymic mouse but not an athymic mouse following an *R. akari* infection (Kenyon and Pedersen, 1980). However, in SCID mice antibodies do protect against *R. conorri* infection (Feng et al., 2004). Therefore, antibodies to play a role in mediating protection however cellular immunity is also important.

In murine and preclinical animals studies cellular immunity is known to play an essential role in protection against rickettsial infections, with both CD4+ and CD8+ T cell-deficient mice demonstrating increased susceptibility to infection with TG, SFG, and *O. tsutsugamushi* (Walker et al., 2001; Moderzynski et al., 2016; Xu et al., 2017). In addition to providing help with B cell production of antibodies, CD4+ T cells release proinflammatory effector molecules, such as IFNγ and TNFα, during infection. Both cytokines have been shown to play an important role in host protection from *R. conorii* and *R. typhi* with depletion of either IFNγ or TNFα leading to enhanced susceptibility in infected C3H/HeN mice (Feng et al., 1994; Moderzynski et al., 2016). One of the ways these cytokines influence the outcome of rickettsial infection is by directly acting on macrophages and neutrophils to enhance their bacterial activity by inducing the expression of reactive nitrogen species (Salim et al., 2016).

Given the intracellular replication of rickettsiae, CD8+ T cell-mediated cytotoxic killing of infected cells is required for bacterial clearance. Transgenic mice that lack the cytotoxic T cell potential (C57Bl/6 Perforin -/-) have been shown to demonstrate increased susceptibility and lethality to *R. australis* (TRG) infection compared with both wild-type (WT) and IFNγ knock out (KO) mice (Walker et al., 2001). However, surprisingly Perforin KO mice in a BALB/c background are equally resistant to *R. typhi* as WT mice (Moderzynski et al., 2017). These findings suggest cytotoxic activity might be more important for protection against some species of rickettsiae than others. However, these results are confounded by the different susceptibility of mice to various rickettsiae where BALBc mice are considered resistant (Eisemann et al., 1984). These studies and the ones above argue that our strategy to provide a Th1 CD4+ T cell, CD8+ cytotoxic T cell, and pathogen specific antibody response should confer the best protection. Another cytotoxin released by CD8+ T cells is Granzyme B, which triggers caspase-mediated apoptosis of infected cells. During infection with *R. typhi* or *R. conorii*, antigen-experienced CD8+ T cell (CD44^high^) numbers are highest around 7-10 days post-infection (Walker et al., 2001; Caro-Gomez et al., 2014a). This time point also coincides with the beginning of bacterial clearance from infected tissues at which point effector-like memory CD8+ T cells (CD27^low^CD43^low^) increase their expression of Granzyme B (Caro-Gomez et al., 2014a). As with CD4+ T cells, stimulated IFNγ release by CD8+ T cells is another strong correlate of protection against rickettsial infections. Mice lacking IFNγ (IFNγ-/-) or its receptor (IFNGR-/-) often demonstrate enhanced susceptibility to rickettsiosis (Moderzynski et al., 2017; Burke et al., 2021). Collectively these data suggest that polyfunctional T cells play a vital role in host defense against rickettsial infections.

Initially, antibody responses were identified as an important component of protection afforded by early *Rickettsia* vaccines based on killed whole cells (Dupont et al., 1973; Mason et al., 1976; Anacker et al., 1985). However, it is now known that antibodies play only a minor role in protection during primary infection and they are not cross-reactive among phylogenetically distant rickettsiae (Feng and Walker, 2003). In contrast, T cells can mediate cross-protection between distantly related rickettsiae, suggesting T cell-mediated immunity is responsible for long-lasting cross-protective immunity (Caro-Gomez et al., 2014a). Thus, T cell antigens should be preferentially considered for inclusion in future vaccines against rickettsiae to provide cross-protection and should be combined with specific B cells antigens identified using the novel techniques described in this review. Identification of novel antibody antigens using bioinformatics and machine-learning may also afford the ability to identify cross-reactive epitopes.

## Typhus group vaccines

The first vaccines against TG rickettsia were developed in the 1920s and pioneered by Polish zoologist Rudolf Weigl. His WCV was produced by isolating the midguts of lice infected with *R. prowazekii*, homogenizing them into a paste, and heat-inactivating (Polak et al., 2022). A total of 30-100 lice were required to produce a single vaccine dose, which provided significant protection in a guinea pig model of infection. His vaccine was later appropriated by the Nazi Party of Germany and used to immunize German soldiers during World War II. However, Weigl is credited with intentionally reducing the efficacy of vaccine doses destined for German soldiers and secretly delivering thousands of doses to Jewish prisoners in ghettos and concentration camps across Poland. Similar WCV derivatives were prepared from isolating *R. prowazekii* from infected rabbit lungs or propagated in yolk sacs (Cox vaccine) before formalin inactivation (Walker, 2009). However, inactivated WCVs were only capable of reducing the severity and duration of disease rather than preventing infection (Dupont et al., 1973). Additionally, there were concerns over the safety of these vaccines due to adverse reactions at the injection site and hypersensitivity to yolk-sacs. The adverse reactions were eliminated in humans by preparing the formalin inactivated WCV in chicken or duck embryo cell culture and showed better protection in animal models however this vaccine was never licensed (Kenyon et al., 1975; Ascher et al., 1978). It is our opinion that WCVs will not provide the best protection because they are not efficient at stimulating CD8+ cytotoxic T cell responses.

Inactivated WCVs were eventually replaced by live attenuated vaccines. In the 1930s, an attenuated strain of *R. typhi* (Casablanca) was created by isolating bacteria from infected guinea pig tissues and treating them with 5% ox bile (previously used in an attenuated dengue vaccine). The resulting vaccine provided heterologous protection against *R. typhi* and *R. prowazekii* (Dyer, 1943). However, differences in bacterial tissue burden between infected animals led to issues regarding quality control and batch-to-batch variation. One of the most successful live attenuated vaccines against TG rickettsia comes from the *R. prowazekii* Madrid strain. Originally termed Madrid 1 after a patient who died from typhus in Madrid during 1941 epidemic, the strain was passaged 11 times through chick embryos and was renamed Madrid E (Richards, 2004). Avirulence of *R. prowazekii* Madrid E is maintained by a mutation in the methyltransferase responsible for methylation of surface proteins (Wei-Mei et al., 1992). The vaccine was originally purified from desiccated yolk-sacs and was demonstrated to elicit protection in guinea pigs without resulting in any recoverable bacteria. Clinical studies of *R. prowazekii* Madrid E strain demonstrated robust immunity against virulent *R. prowazekii* Naples and Breinl strains that lasted at least 5 years post-immunization (Fox, 1955). Several years later it was determined that some adverse reactions were likely a result of yolk-sac contaminants and endotoxin, so modifications were made to the manufacturing. The Madrid E vaccine is no longer in use due to the persistence of some adverse events, uncertainty over the mechanism and stability of attenuation, and concerns over the potential for unrestricted growth in immunocompromised individuals (Zhang et al., 2006).

Most subunit vaccine candidates against rickettsiae are surface-exposed antigens recognized by antibodies to enhance bacterial uptake into phagocytes. However, given the significance of cellular immunity in host protection from rickettsial disease, efforts to identify vaccine candidates have focused on discrete antigens that trigger T cell engagement. Although very few rickettsial antigens have been identified, the most promising belong to the surface cell antigen autotransporter family (Sca 0-5) involved in bacterial adherence and cell uptake. Of particular interest is outer membrane protein B (OmpB/Sca5), which is recognized by convalescent serum from laboratory animals and patients previously infected with *R. typhi* (Dasch and Bourgeois, 1981). Additionally, T cells from *R. typhi* infected patients secrete IL-2 and IFNγ in response to macrophages expressing fragments of *R. rickettsii* OmpB, suggesting recognition of peptides presented by MHCI by CD8+ T cells and cross-reactivity of T cells to conserved OmpB epitopes between TG and SFG rickettsiae (Dzul-Rosado et al., 2017). Guinea pigs vaccinated with OmpB from *R. typhi* are protected from homologous challenge (Dasch et al., 1984).

Using a reverse vaccinology approach, Caro-Gomez et al. identified a collection of novel antigens (RP403, RP598, RP739, and RP778) from *R. prowazekii* based on the presence of CD8+ T cell epitopes (Caro-Gomez et al., 2014b). These antigens were recombinantly expressed in SCEV 4-10 cells for presentation by MHCI and were then used to immunize C3H/HeN mice. Immunization resulted in an antigen-specific CD8+ T cell response that produced IFNγ and granzyme B, which protected mice from a lethal heterologous challenge with *R. typhi* (Caro-Gomez et al., 2014a). We hope that novel strategies to identify B and T cells antigens developed during the Sars-Cov-2 pandemic are used to further identify potentially protective epitopes.

## Spotted fever group vaccines

Like the first TG vaccines, early SFG vaccines consisted of fixed WCV material. In 1925, Spencer and Parker prepared the first RMSF vaccine by isolating *R. rickettsii* from the intestines of infected ticks and inactivating the material in phenol (Spencer and Parker, 1925). While two doses of this vaccine were able to elicit strong protection in rhesus macaques, the vaccine was only able to reduce the severity of illness in humans rather than prevent infection. Subsequent phenol and formalin-treated suspensions of *R. ricketsii* from yolk sacs (Cox method) were produced but they were only effective at delaying the onset of symptoms and shortening the duration of illness. Improvements to SFG vaccines were eventually made using *R. rickettsii* cultures grown in chick embryo fibroblasts and purified by sucrose gradient. We again postulate that WCVs will not be protection because they are not efficient at stimulating CD8+ cytotoxic T cell responses.

Of the SFG immunogens identified experimentally, outer membrane proteins A (OmpA/Sca0) and B (OmpB/Sca5) have been studied most. Immunization of guinea pigs with recombinant OmpA from *R. rickettsii* has been shown to elicit strong protection from homologous challenge and partial protection from heterologous *R. conorii* challenge (Vishwanath et al., 1990). DNA vaccination using fragments of OmpA (OmpA703–1288, OmpA755–1301 or OmpA980–1301 or OmpA1644–2213) or OmpB (OmpB451–846 or OmpB754–1308) from *R. conorii* have also been used for immunization. A priming dose using plasmid DNA followed by boost immunization with the corresponding recombinant peptide induced a significant IFNγ response in T cells upon *in vitro* recall with *R. conorii* WCV. This boosting strategy was also successful in protecting mice from lethal challenge with *R. conorii* (Crocquet-Valdes et al., 2001). Interestingly, heterologous DNA immunization with plasmids encoding fragments of OmpA and OmpB (OmpA703–1288 or OmpA1644–2213 with OmpB451–846 or OmpB754–1308) was also protective against lethal challenge with *R. conorii* and shown to be more immunogenic than either vaccine alone (Díaz-Montero et al., 2001). Antibodies likely play a significant role in OmpA/B mediated protection given passive immunization of antigen-specific antibodies into guinea pigs and immunodeficient SCID mice protects animals from a lethal *R. rickettsii* challenge (Feng et al., 2004). Additionally, OmpA/B specific antibodies have been shown to facilitate opsonophagocytosis of *R. conorii*, inhibit adherence of *R. rickettsii* to host cells, and mediate complement-dependent killing of SFG rickettsia. Each of these antibody-mediated mechanisms may contribute to protection against infection.

Another antigen of interest is YbgF, a Tol-Pal system protein identified as a major seroreactive surface exposed protein (Qi et al., 2013b). Immunizing C3H/HeN mice with recombinant YbgF resulted in the proliferation of IFNγ secreting CD4+ and CD8+ T cells and fewer bacteria in the spleen, liver, and lungs following infection with *R. rickettsia* (Gong et al., 2015a). Similarly, C3H/HeN mice immunized with recombinant YbgF from *R. heilongjiangensis* (the causative agent of Far-Eastern Spotted Fever; FESF) results in a CD4+ T cell response that limits the bacterial burden upon infection with homologous bacteria (Qi et al., 2013a). Vaccination with another component of the *R. rickettsii* Tol-Pal system, TolC, was also shown to be immunogenic but less efficient at restricting rickettsial burden and impairment in the spleen, liver, and lungs than YbgF (Gong et al., 2015a).

Other immunogenic proteins that have been used for experimental vaccination against SFG rickettsiae include Adr1, Adr2, OmpW, and Porin-4. C3H/HeN mice immunized with recombinant Adr1, OmpW, or Porin-4 lead to a reduced bacterial burden in tissues upon challenge with *R. rickettsia* (Gong et al., 2014a). Similarly, immunization with recombinant Adr2 protects mice from *R. rickettsii* infection and leads to enhanced production of IFNγ by CD4+ T cells and TNFα by CD8+ T cells (Gong et al., 2014b). *In vitro* neutralization assays revealed that sera from mice immunized with recombinant Adr1, Adr2, or OmpW reduced *R. rickettsii* adherence to and invasion of vascular endothelial cells. In many instances, combination vaccines provide the most promising outcome from infection. One such example is a combination of recombinant Adr2 with OmpB, which results in enhanced protection against *R. rickettsii* infection than either one alone, resulting in a stronger IFNγ response and fewer detectable bacteria (Gong et al., 2015b).

## *Orientia* spp. vaccines

Early *O. tsutsugamushi* vaccines were WCV derivatives purified from the lungs of infected cotton rats and fixed in formalin. The protection provided by this vaccine in humans was initially based on 15 out of 16 individuals developing mild disease, which led to large-scale production of the vaccine towards the end of World War II (Walker, 1947). Similarly, studies with this vaccine showed it provided only partial protection of mice against infection, resulting in a milder disease outcome (Bailey et al., 1948). A human field study performed later in Japan showed that there was no demonstrable protection afforded by this vaccine (Berge et al., 1949). One of the drawbacks to using formalin-inactivated vaccines is antigen modifications resulting from formalin cross-linking. An alternative approach using gamma-irradiated *O. tsutsugamushi* was found to protect mice against a lethal challenge with a homologous strain over 12 months (Eisenberg George and Osterman Joseph, 1978). However, protection against heterologous strains appeared to wane rapidly and was not significant after 6 months.

Strain diversity among *O. tsutsugamushi* is one of the biggest challenges to vaccine development, as noted by cases of reinfection and the lack of serological cross-reactivity in human cases (Philip, 1948; Kuwata, 1952). Sequence analysis of known immunodominant antigens, including type-specific antigen (TSA) 56 and surface cell antigens (*sca*), has shown variable homology and levels of conservation among different *O. tsutsugamushi* strains (Ruang-Areerate et al., 2011; Ha et al., 2012). Furthermore, comparative genomics of two *O. tsutsugamushi* strains (Boryong and Ikeda) revealed a common set of repetitive sequences that have been explosively amplified in both genomes, which resulted in extensive genome shuffling as well as duplications and deletions of many genes. Consequently, natural infection with *O. tsutsugamushi* does not provide long-term protection from reinfection, particularly from different strains. In a human trial involving individuals previously infected with scrub typhus, reinfection with *O. tsutsugamushi* resulted in a similar severity of illness compared to naïve controls (Smadel et al., 1950). The original strain responsible for infection in most of the volunteers was believed to have been different from the one used in the study (Gilliam) and was therefore likely a heterologous challenge. However, for one of the volunteers it was known that the original infection, 3 years prior, was caused by *O. tsutsugamushi* Gilliam and this individual developed no other symptoms besides erythema at the site of inoculation. In this instance, heterologous immunity may be very short-lived while homologous immunity may last longer.

Characterization of *O. tsutsugamushi* immunodominant antigens by reactive convalescent serum with bacterial lysates revealed five major proteins were common to three strains (Eisemann and Osterman, 1981). They were identified as TSA 22, 47, 56, 58, and 110 based on their molecular weight. Of these five antigens, TSA56 has been regarded as one of the more favorable vaccine targets due to conserved epitopes among different strains of *O. tsutsugamushi* and its role in the attachment and invasion of host cells (Cho et al., 2010). Mice immunized with TSA56 not only generate a robust humoral response, including neutralizing antibodies, but elevated IFNγ and IL-2 production, associated with T cells. These mice were also shown to have measurable protection against a homologous challenge with *O. tsutsugamushi* Boryong strain (Choi et al., 2014). Furthermore, a novel recombinant antigen comprised of conserved regions of TSA56 was shown to elicit protection against both homologous and heterologous lethal challenges (Kim et al., 2019). The authors also demonstrated protective immunity could be adoptively transferred using CD4+ or CD8+ T cells from immunized mice, whereas immune B cells failed to do so. Thus, providing further evidence of the importance cellular immunity against conserved epitopes plays in protective immunity against scrub typhus. Other successful studies demonstrating a protective role of TSA56 include a fusion with maltose-binding protein (MBP) and a truncated version (r56). In both instances, immunized mice exhibited high titer antigen-specific antibodies and cellular immune responses that resulted in protection from a homologous, but not heterologous lethal challenge (Seong et al., 1997; Chattopadhyay and Richards, 2007).

The TSA47 outer membrane protein is another major antigen that has been considered for vaccination. Polyclonal T cells derived from infected mice are highly stimulated in the presence of TSA47 and antigen-specific antibodies are cross-reactive with at least 8 different strains of *O. tsutsugamushi* (Oaks et al., 1989; Hickman et al., 1993). Upon immunizing mice with a DNA construct containing TSA47, outbred CD-1 mice were found to produce polyfunctional splenocytes secreting IFNγ, IL-4, and IL-13 in addition to a strong antibody response (Chattopadhyay and Richards, 2007). In challenge studies using TSA47 as the immunizing antigen delivered intranasally, mice developed high antibody titers, including IgG and IgA in bronchoalveolar lavage (BAL) fluid, as well as cellular immunity correlative with a Th1 response (Choi et al., 2017; Park et al., 2021). In both studies, mice were significantly protected against a lethal challenge with *O. tsutsugamushi* Boryong. A fusion protein of TSA47 with TSA56 from *O. tsutsugamushi* Karp was evaluated for its efficacy in protecting mice against a homologous challenge. In this study, the fusion protein was found to provide greater protection than when either protein was given individually (Yu et al., 2005). However, protection was only partial (50%) and the efficacy of a TSA47-56 fusion protein against heterologous challenge remains to be determined.

Other immunogenic proteins of interest from *O. tsutsugamushi* are the autotransporter proteins ScaA, ScaC, ScaD, and ScaE. Each of these antigens has been shown to cross-react with convalescent serum with ScaA identified as the most reactive (Ha et al., 2012). Immunizing mice with ScaA resulted in enhanced protection against both homologous (Boryong strain) and heterologous (Karp and Kato strains) *O. tsutsugamushi* challenge when combined with TSA56 (Ha et al., 2015). In a separate study from the same group, ScaA was coupled to zinc oxide nanoparticles that were taken up by DCs *in vitro* and induced protective immunity in mice. Immunized mice developed antibodies against ScaA as well as IFNγ producing CD4+ and CD8+ T cells.

Given that natural immunity to *O. tsutsugamushi* wanes over time and there is significant antigenic diversity among strains, it is likely any successful vaccine against scrub typhus will need to overcome these challenges. Currently, the number of antigens identified as potential *O. tsutsugamushi* vaccine targets remains limited, and further studies will need to rely on identifying antigens based on T cell epitopes in addition antibody cross-reactivity. Antigens that elicit strong cellular responses, including IFNγ secreting CD4+ and CD8+ T cells, are likely necessary for protection against rickettsial diseases. Memory cells that have a bimodal distribution consisting of both effector memory and central memory phenotypes will help to overcome issues of waning immunity. Lastly, vaccines containing fusion proteins or combinations of multiple antigens are likely to improve the breadth of immunity across different strains to support heterologous protection, however, it is unknown how many fusion partners will be needed. Again, we hope that novel strategies to identify B and T cells antigens developed during the Sars-Cov-2 pandemic are used to further identify potentially protective epitopes.

## C. burnetii

### Bacteria and disease overview

*C. burnetii* is a Gram-negative obligate intracellular bacterium and the causative agent of the zoonotic disease query fever (Q fever) (Van Schaik et al., 2013). Q fever can present either as an acute self-limiting febrile disease or as a chronic disease most commonly associate with endocarditis (Maurin and Raoult, 1999). Transmission usually occurs after the inhalation of contaminated aerosols. Once inside the lungs *C. burentii* are taken up by alveolar macrophages where they grow and divide in a compartment like a lysosome termed the *Coxiella*-containing vacuole (CCV). *C. burnetii* is listed as a Category B select agent and therefore a safe and efficacious vaccine should be developed.

### CoPs

Understanding the mechanisms of immunity to *C. burnetii* can guide the development of protective vaccines against this pathogen. Evaluation of adaptive immune responses after infection or vaccination in humans and laboratory animals indicates that while both cellular and humoral immune responses play a role in protection, cell-mediated immunity is essential for resolution of infection. Humans with acute Q fever develop serum IgM, IgG, and IgA titers against *C. burnetii* antigens that persist for long periods after resolution of infection, while those with chronic Q fever have very high phase I IgG and significantly lower phase II IgM titers (Worswick and Marmion, 1985; Buijs et al., 2021). In mice, experiments using adoptive transfer of immune serum show that serum IgG provides some protection against infection but does not fully control bacterial dissemination and growth. Comparisons of the humoral responses to protective phase I WCVs and non-protective phase II WCVs in mice indicate that IgG targeting phase I lipopolysaccharide may play a significant role in protection against *C. burnetii* (Zhang et al., 2007). *C. burnetii* phase I LPS has been shown to shield surface antigens from antibody binding through steric hindrance, prevent dendritic cell activation by blocking toll-like receptor (TLR) ligands, and resist complement-mediated serum killing (Hackstadt, 1988; Vishwanath and Hackstadt, 1988; Shannon et al., 2005). Abinanti and Marmion showed that treating *C. burnetii* with immune serum from rabbits exposed to phase I *C. burnetii*, but not serum from rabbits exposed to phase II, inhibited infection in mice (Abinanti and Marmion, 1957). *In vitro* experiments showed that treatment of *C. burnetii* with serum containing anti-*C. burnetii* IgG potentiates phagocytosis and destruction of bacteria by macrophages *in vitro* (Kishimoto and Walker, 1976). However, the adoptive transfer of immune serum into T cell-deficient, athymic mice had no significant impact on infection compared to mice receiving non-immune serum, indicating that T cell-mediated immunity is essential for the control of *C. burnetii* infection (Zhang et al., 2007).

The role of cell-mediated immunity has been evaluated in both humans and laboratory animals. Measurements of the index of lymphoproliferative response, a measure of lymphocyte proliferation upon exposure to an antigen, in humans after vaccination with a WCV showed positive LSI in 85-95% of vaccinees, while only 35-70% showed positive serum antibody titers against *C. burnetii* (Izzo et al., 1988). In mice, depletion of T cells results in an inability to control pulmonary infection with *C. burnetii* (Read et al., 2010). However, both CD4+ and CD8+ T cells appear to play a role in immunity to *C. burnetii* as immunodeficient mice lacking T cells reconstituted with either CD4+ or CD8+ T cells can control infection (Read et al., 2010).

CD4+ T cells activated through antigen-presentation on MHCII produce IFNγ which enhances phagocytosis and production of reactive oxygen species and reactive nitrogen species by macrophages which are an important target cell of *C. burnetii* infection (Andoh et al., 2007). *In vitro* stimulation of infected monocytes with IFNγ inhibits intracellular *C. burnetii* replication and survival and induces apoptosis (Dellacasagrande et al., 1999). CD4+ T cells appear to play an important role in the control of *C. burnetii* replication, however, *in vivo* mouse studies suggest that CD8+ T cells play a larger role in bacterial clearance. In MHCI and MHCII knock-out (KO) mice, MHCII KO mice infected with *C. burnetii* can clear infection, MHCI KO mice develop persistent infections (Buttrum et al., 2018). The role of CD8+ T cells in the control of *C. burnetii* infection is not fully understood, however, perforin may be an important mediator of protection as perforin-deficient mice develop significantly more severe splenomegaly than wild-type mice during challenge (Buttrum et al., 2018). Overall, an effective vaccine for *C. burnetii* will likely need to produce strong cell-mediated memory from both CD4+ and CD8+ T cells to control intracellular replication and bacterial clearance as well as humoral immunity to enhance vaccine efficacy by neutralizing bacteria before infection of host cells. The current WCVs for *C. burnetii* fail to elicit CD8+ T cell responses and therefore could be improved upon using strategies described in this review.

## Current vaccine strategies WVC

*C. burnetii* is an obligate intracellular bacterium that is endemic worldwide except for New Zealand and Antarctica and is maintained by persistent infection in sheep, goats, cattle, and camelids, making elimination from the environment extremely difficult (Hilbink et al., 1993; Van Schaik et al., 2013). Because of this, an effective vaccination program is considered essential to reduce the rate of human infections. Although abbatoir workers, farmers, and veterinary professionals working closely with these domestic species are considered at the greatest risk of infection, interest in the development of vaccines against Q fever was initially driven by concern for the protection of military troops. Some of the first isolates of *C. burnetii* were collected from American soldiers who were infected during their deployment to Italy during World War II. To isolate the bacterium, blood samples from infected patients were inoculated into guinea pigs and then cultured in embryonated yolk sacs. This work produced several strains of *C. burnetii* including the Henzerling strain which was later used for the production of the first formalin-inactivated WCV (Robbins and Rustigian, 1946; Snyder et al., 1947).

Early evaluation of this WCV in a guinea pig model of infection showed that it reduced mortality to less than 2% in vaccinated animals compared to 40-80% in the unvaccinated group (Snyder et al., 1947). Administration of this vaccine in humans showed similarly high levels of protection. An 18-month survey of abattoir workers in southern Australia reported no cases of Q fever among 924 workers vaccinated with a single dose of the formalin-inactivated, Henzerling strain phase I vaccine compared to 34 cases in 1349 unvaccinated (control) workers (Marmion et al., 1984). However, early experiments in humans and animals noted local and systemic reactions occurred in some vaccinated individuals (Snyder et al., 1947). These reactogenic responses would continue to be a major barrier to the widespread use of the WCV.

There are currently two licensed formalin-inactivated, WCVs against *C. burnetii*: Q-VAX^®^ (Seqirus, Australia) and Coxevac^®^ (CEVA Sainté Animale, France), administered to humans and ruminants, respectively. Q-VAX^®^ was developed using the phase I Henzerling strain of *C. burnetii* in response to the high rate of infections among abattoir workers in Australia and is only licensed for human use in that country. Clinical trials of Q-VAX^®^ and later retrospective studies showed long-lasting protection of greater than 90% 5 years after vaccination (Marmion et al., 1990; Woldeyohannes et al., 2020). Additionally, the implementation of a vaccination program in Australia in 2002 reduced the incidence of Q fever between 2002 and 2006 by over 50% (Gidding et al., 2009). Coxevac® is similarly a whole cell, formalin-inactivated vaccine derived from the phase I Nine Mile strain and licensed for use in ruminants in Europe. Although Coxevac does not significantly reduce infection rates in ruminants, this vaccine has been shown to lower abortion rates in infected animals and reduce exposure risk to humans by decreasing bacterial shedding through vaginal fluids, feces, and milk (Rousset et al., 2009; De Cremoux et al., 2012).

Inactivated WCVs against *C. burnetii* are proven to be highly effective at reducing infections in humans, but the high rates of local and systemic reactions after vaccination have prevented widespread licensure of this vaccine (Fratzke et al., 2022). Even in early experiments, local induration, erythema, and chronic granulomas with abscesses at the vaccine site were common, and systemic symptoms such as fever, malaise, and anorexia were frequently reported in vaccinated humans with a history of prior exposure to *C. burnetii* (Snyder et al., 1947). Injection site granulomas from the WCV may last for weeks to months and occasionally require surgical excision to resolve (Bell et al., 1964). Similar local reactions to *C. burnetii* WCVs have been reported in ruminants, rabbits, and guinea pigs (Biberstein et al., 1974). To reduce severe reactions to the WCV, humans receiving Q-VAX^®^ must undergo pre-vaccination screening which includes measuring anti-*C. burnetii* antibody titers in serum and intradermal sensitivity testing. This intradermal skin test uses a very low dose of whole cell material injected into the skin, and then local induration and erythema are measured 48 hours later, similar to the tuberculin test for Mycobacteriosis (Lackman et al., 1962). Despite this pre-vaccination screening strategy, adverse responses to the whole-cell vaccine are still common, with local and systemic reactions reported in 98% and 60% of seronegative, skin test-negative veterinary students, respectively, in a 2018 survey (Sellens et al., 2018).

**Figure 3:**
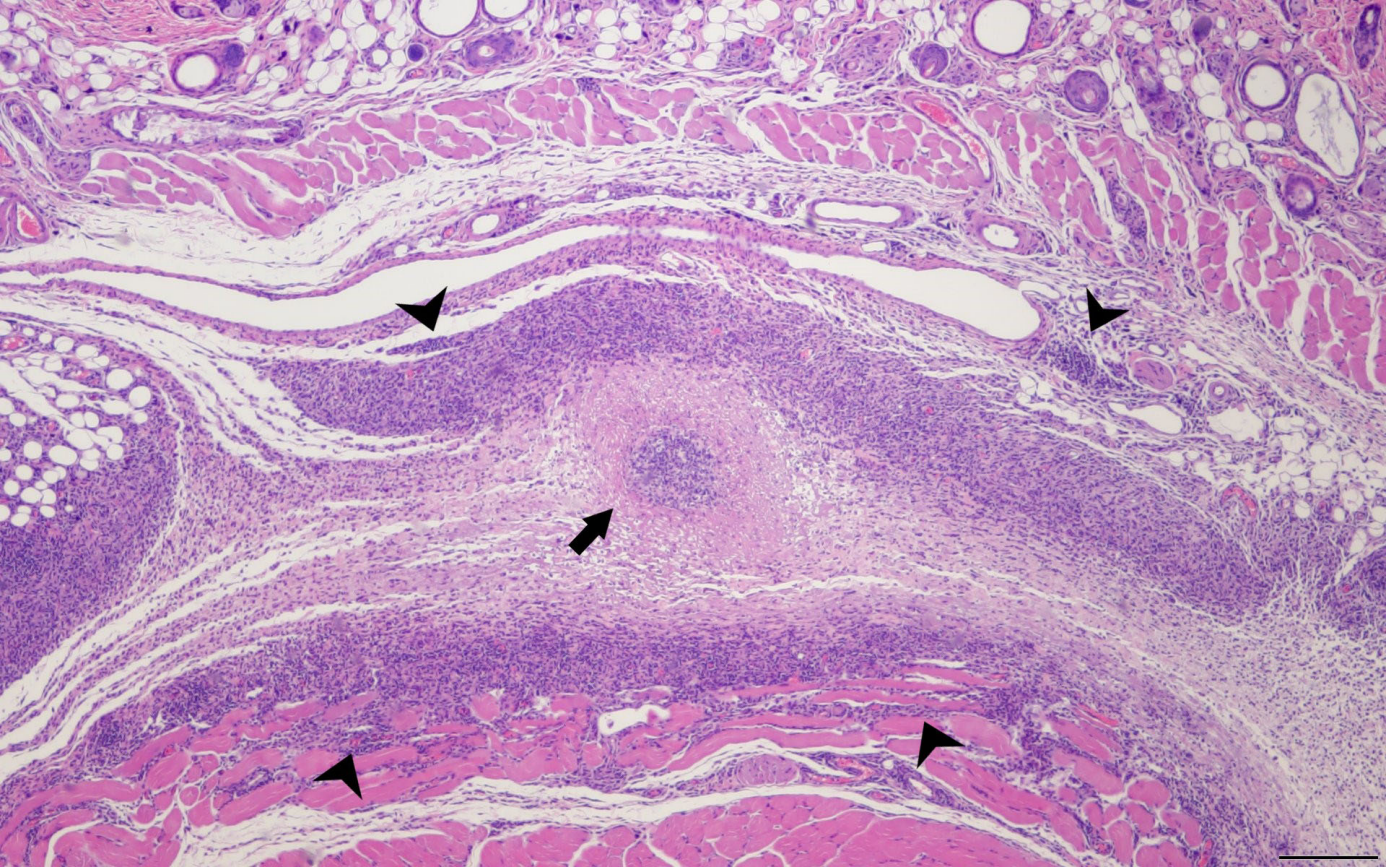
Delayed Type Hypersensitivity Response in the Mouse Model. SKH1 mice were infected with *C. burentii* and then the infection was resolved before WVC prepared from NMI was injected intramuscularly. The skin sections were removed 2 weeks later and sent for H&E staining. This is a representative image. A central abscess (arrow) surrounded by lymphohistiocytic inflammation (arrowheads) within the subcutaneous tissue. Hematoxylin and eosin, 4x magnification, bar=200 µm.

The pathogenesis and underlying cause of *C. burnetii* WCV reactogenicity are poorly understood. Some early research speculated that phase I LPS is the cause of local reactogenic responses, but more recent studies have disproven this hypothesis (Kazár et al., 1982; Long et al., 2021; Fratzke et al., 2021b). These local and systemic reactions are more frequent and severe in individuals with antibody titers against *C. burnetii* from prior exposure and histopathologic evaluation of vaccine site reactions show infiltration of macrophages, neutrophils, and lymphocytes with central abscesses suggesting that *C. burnetii* WCV reactions are a granulomatous type IV hypersensitivity response (Bell et al., 1964; Kazár et al., 1982; Wilhelmsen and Waag, 2000). Type IV hypersensitivities have a delayed onset and are driven by memory CD4+ and CD8+ T cells. Recently, (Fratzke et al., 2021a) showed that in sensitized mice, local and systemic reactions to the whole-cell vaccine are characterized by an increase in IFNγ and IL17a+ CD4+ T cells, indicating a Th1/Th17-mediated hypersensitivity (Fratzke et al., 2021a) (**Figure 3**). However, research into the mechanisms behind the efficacy of the WCV suggest that while anti-*C. burnetii* antibodies provide some immunity, T cell-mediated immune memory is critical for vaccine-induced protection against infection (Zhang et al., 2007). Thus, novel vaccine strategies against *C. burnetii* must provide a balanced immune response to maintain protective immunity while reducing adverse reactions.

## Early alternatives to the *C. burnetii* WCV

Several alternative *C. burnetii* vaccines have been tested over the decades since the development of the WCV, including phase II inactivated, modified live, and solubilized and residual antigen vaccines (Robinson and Hasty, 1974; Kazár et al., 1982; Williams et al., 1992; Zhang et al., 2007). Serial passages of *C. burnetii* through embryonated yolk sacs results in attenuation of the bacteria, termed phase variation. A large chromosomal deletion occurs during this passage resulting in the truncation of the LPS (Beare et al., 2018). Phase I *C. burnetii*, isolated from infected animals or humans, is highly virulent and infectious while the phase II form, isolated after multiple passages through embryonated yolk sacs, shows a marked reduction in infectivity except in immunocompromised humans and animals (Van Schaik et al., 2017). This makes phase II *C. burnetii* much safer to manufacture for use in vaccines and allows for work under biosafety level 2 conditions instead of the high containment required for phase I bacteria. However, a formalin-inactivated, WCV derived from phase II *C. burnetii* produces a marked reduction in protective efficacy compared to phase I-derived vaccines (Stoker and Fiset, 1956). This phase variation was later determined to be due to alteration in the LPS expressed on the surface of *C. burnetii* and later studies showed that phase I LPS is an essential component for the efficacy of the WCV (Schramek et al., 1983; Zhang et al., 2007).

Originally developed in the 1960s and 70s, a solubilized antigen vaccine was derived from phase I *C. burnetii* using trichloroacetic acid (TCA) extraction (Kazár et al., 1982). This vaccine showed >90% protective efficacy in mice and guinea pigs challenged by intraperitoneal injection with phase I *C. burnetii* and, in humans vaccinated with a two-dose regimen, positive sera was detected in 74.4% of volunteers at 5 weeks post-vaccination. The TCA vaccine also resulted in overall high rates of local reactions in humans, ranging from 27.9% to 63.9%, but a decrease in the severity of these reactions compared to the WCV (Kazár et al., 1982). A live attenuated vaccine, M-44, was developed in Russia in the 1960s by serial passage of the Grita strain of *C. burnetii* forty-four times through embryonated chicken eggs. M-44 showed a marked loss of virulence when inoculated in guinea pigs and mice (Marmion, 1967). Although this vaccine provided significant protection against Q fever in animal and human experiments and showed reduced local and systemic reactions, concerns about the safety of attenuated *C. burnetii* vaccines prevented their general use (Robinson and Hasty, 1974). Indeed, one study reported that the M-44 vaccine could not only persist in tested animals for long periods, but also induce inflammation in the liver, spleen, and heart of vaccinated guinea pigs (Johnson et al., 1977).

Perhaps the most well-developed novel vaccine strategy to date is the chloroform:methanol residue (CMR) vaccine developed in the 1980s by the Rocky Mountain Laboratories in the United States. This method separated whole cell material into a soluble fraction, mainly composed of lipids, and a precipitate or residual fraction, composed of LPS, proteins, and peptidoglycans, which was developed as the CMR vaccine (Williams and Cantrell, 1982; Williams et al., 1992). A prime-boost regimen of the CMR vaccine provided similar protection compared to WCV in guinea pigs administered 1.4x10^10^ phase I *C. burnetii* by intraperitoneal injection (Williams et al., 1992). More recently, a phase I clinical trial of the CMR vaccine produced significant anti-*C. burnetii* IgG titers and effective T cell priming. Though 75% and 65% of the 20 participants developed local reactions after the prime and boost vaccinations, respectively, these local reactions appeared less persistent than those reported with the WCV, peaking at approximately 2 to 3 days post-vaccination and resolving by 7 days post (Waag et al., 2008). However, further clinical safety and efficacy trials of this vaccine have not been performed.

## Current research for novel *C. burnetii* vaccines

Since the turn of the millennium, two more events prompted renewed interest in the development of non-reactogenic vaccines against *C. burnetii*. Between 2007 and 2010, a large outbreak of more than 4,000 cases of Q fever occurred in humans in the Netherlands linked to infections among several dairy goat farms. An emergency vaccination program using Q-VAX^®^ was implemented to help control the outbreak but was hindered by the need for pre-vaccination screening. 22% of high-risk individuals were excluded from the vaccination program due to positive skin test or serology (Isken et al., 2013). Additionally, U.S. military deployment to rural areas in developing countries in the early 2000s has led to increased exposure risk in military personnel as evidenced by sporadic infections and seroconversion rates of 2.1-7.2% (Gleeson et al., 2007; Royal et al., 2013). These exposure risks as well as *C. burnetii*’s status as a potential bioweapon substantiate the need for a safe and effective vaccine for military troops and other at-risk populations.

In some recent studies, novel adjuvants have facilitated the development of new subunit and solubilized antigen vaccines for *C. burnetii.* The increased availability of the number and types of vaccine adjuvants have not only allowed researchers to overcome the decreased protective efficacy of subunit vaccines but also refine immune responses. A *C. burnetii* subunit vaccine using different combinations of TLR agonist adjuvants was effective in protecting mice and guinea pigs against aerosol challenge. While some adjuvant combinations still incited vaccine site granulomas, one adjuvant combination demonstrated a significant reduction in local adverse responses (Gilkes et al., 2020; Fratzke et al., 2021b). In a separate study, a solubilized protein vaccine derived from phase II *C. burnetii* combined with the TLR9 agonist adjuvant CpG, which stimulates strong T cell memory responses, showed significant protection against aerosol challenge in mice, guinea pigs, and non-human primates and marked reduction in hypersensitivity responses in sensitized guinea pigs (Gregory et al., 2021). This strategy is appealing because it does not rely on cultivation of *C. burnetii* in a high-containment BL3 laboratory.

Advances in genetics and molecular biology have also allowed for the creation of new mutant strains of *C. burnetii* which do not express potentially reactogenic antigens. A mutant strain of phase I *C. burnetii* lacking the type IV secretion system, *C. burnetii dot/icm*, was recently tested as an alternative WCV (Long et al., 2021). This mutant WCV provided similar protection to the wild-type WCV in a guinea pig challenge experiment. Although evaluation in sensitized guinea pigs showed mild reductions in local erythema and induration, histologic evaluation of vaccination sites did not reveal a significant reduction in the severity of local inflammation compared to the wild-type WCV (Long et al., 2021). This strategy was refined by creating a *dot/icm* Dugway mutant, a *C. burnetii* isolate that is not virulent in animal models including mice and guinea pigs in its wild-type form (Russell-Lodrigue et al., 2009; Tesfamariam et al., 2022). Vaccination with *dot/icm* Dugway provides similar protection to WCV and reduced the reactogenicity (Tesfamariam et al., 2022). These recent attempts to produce safer, novel vaccines have yet to be tested in human clinical trials. Although these formulations are ready for phased trials, we hope that novel strategies to identify B and T cells antigens developed during the Sars-Cov-2 pandemic are used to further identify potentially protective epitopes for testing in novel delivery platforms.

## Anaplasmataceae

### Bacteria and disease overview

The incidence of tick-borne diseases in the United States has increased over the past two decades. This includes more prevalence of acute febrile tick-borne diseases in humans caused by many species of the genera *Ehrlichia* and *Anaplasma* which are mainly transmitted by Lone Star ticks and Blacklegged ticks respectively (Rikihisa, 2010) (**Figure 2**). This increased burden and the associated morbidity highlights the need for safe and efficacious vaccines. The most common causes of human infections are *Ehrlichia chaffeensis* which causes human monocytic ehrlichiosis (HME) and *Anaplasma phagocytophilum* which causes human granulocytic anaplasmosis (HGA) (Ismail and Mcbride, 2017). Although there are other species in each genus that can cause infections in humans, we will focus on *E. chaffeensis* and *A. phagocytophilum*. Both cause flu-like illnesses that often go misdiagnosed, thus, the actual incidence of the disease is much higher. The therapy of choice is the broad-spectrum antibiotic doxycycline, which is only effective if initiated early on (Hamburg et al., 2008). Vaccination therefore would be the most effective intervention to prevent the spread of these emerging infectious diseases. The major challenges to developing efficacious vaccines for *E. chaffeensis* and *A. phagocytophilum* include antigen variability and these bacteria (like most obligate intracellular pathogens) subvert the innate and adaptive immune responses which makes vaccine development even more challenging (Salje, 2021). Finally, the major challenge for all obligate intracellular pathogens remains the lack of genetic tools for manipulation (Mcclure et al., 2017). This lack of genetic tools has hampered the identification of pathogenic mechanisms, which is important to designing effective vaccines. Again, we hope that novel strategies to identify B and T cells antigens developed during the Sars-Cov-2 pandemic are used to further identify potentially protective epitopes.

### CoPs

Rational vaccine development can resolve some of these issues by deciphering the immune correlates of protection from infection-resolved cases. Strong immunity can be developed in both humans and animals that have recovered from Ehrlichiosis and Anaplasmosis. Understanding what drives immunity during natural infection in animal models has provided many clues for vaccine strategies for both HME and HGA. A Th1-mediated cellular response seems to be important for the clearance of infection. The first clue that IFN-γ was important for clearance of infection came from *in vitro* studies with human monocytes demonstrating that pre-treatment with IFN-γ inhibited infection with *E. chaffeensis* (Barnewall and Rikihisa, 1994). Shortly thereafter several mouse models of infection were developed including *Ixodes ovatus* ehrlichia (IOE) which demonstrated that IFN-γ production by CD4 T cells was essential for resolving infection (Bitsaktsis et al., 2004). Studies in IFN-γ -/- mice demonstrated that Th1 skewed response with IFN-γ production also facilitated bacterial clearance of *A. phagocytophilum* where it is involved in early infection control but dispensable at later timepoints (Akkoyunlu and Fikrig, 2000). However, it was later determined that although CD4+ T cells are required for pathogen clearance Th1 cytokines are dispensible and therefore the mechanism of protection remains to be identified (Birkner et al., 2008). This highlights the paradigm that Th1 CD4 T cells are required to resolve infections with most intracellular bacterial pathogens, however, they do not explain all of the protective mechanisms which occur through unknown mechanisms (Thakur et al., 2019).

The importance of these cellular responses goes against to dogma that most vaccines historically have aimed to induce neutralizing antibodies. This strategy is not as effective for intracellular pathogens, where antibodies can induce uptake and infection of cells by a mechanism termed antibody-dependent enhancement (ADE) which can exacerbate natural infection (Thomas et al., 2006). Although this is possible, high antibody titers are seen after the resolution of natural infection with both *E. chaffeensis* and *A. phagocytophilum* in humans (Lotric-Furlan et al., 2001; Aguero-Rosenfeld et al., 2002). Other clues to the protective nature of sera have been demonstrated in animal models of infection. For example, although wild-type inbred mice are relatively resistant to *E. chaffeensis* infection, SCID mice provide a relevant animal model of infection. Passive transfer of sera from *E. chaffeensis* infection resolved wild-type mice to SCID mice before or after inoculation with *E. chaffeensis* provides a measurable level of protection (Winslow et al., 2000). However, this study also confirmed that T cells are required for complete clearance of infection highlighting again the importance of cellular response in protection (Winslow et al., 2000). The mechanism of antibody-mediated protection for *E. chaffeensis* was further elucidated to depend on complement and FcγR-mediated phagocytosis (Yager et al., 2005). Individuals infected with *A. phagocytophilum* have an early IgG antibody response to 2 proteins at 40 and 44-kDa respectively suggesting an important role for CD4 T cell help (Jw et al., 1997). This class switching in humans plus the evidence that CD4 T cells are important for clearance of infection, demonstrates that both humoral and cell-mediated immunity are involved. Studies in mice have also demonstrated that humoral response to *A. phagocytophilum* provides partial protection (Sun et al., 1997). Antibody responses must be against surface-exposed proteins to elicit neutralizing effects. However, it is more convoluted with intracellular bacteria where it is common that serum transferred to athymic mice is not protective indicating that neutralization alone may not confer protection (Humphres and Hinrichs, 1981; Rhinehart-Jones et al., 1994). All this information from animal models and human infections demonstrates that both humoral and cell-mediated responses will be necessary to provide protection and therefore any vaccine candidate must elicit both responses.

## *Ehrlichia* spp. vaccines

One of the major challenges for vaccine development for obligate intracellular bacteria was the choice of antigen. As *Ehrlichia* spp. lack lipopolysaccharides; glycoconjugate strategies are not valid making identification of protein antigens necessary. Until very recently the number of immunoreactive proteins identified was only a handful including several tandem repeat proteins (TRPs) like gp120 (Luo et al., 2009), an ankyrin protein Ank200 (Luo et al., 2010), and the major outer membrane protein (OMP) (Ohashi et al., 1998). However, advancements in next-generation sequencing in recent years have allowed the discovery of antigenic proteins using ANTIGENpro which identifies potentially protective antigens for a humoral response (Luo et al., 2020, 2021). The *E. chaffeensis* antigens identified in these studies were mostly hypothetical secreted proteins that require the native protein structure to be conserved for antibody recognition, indicating that they are not linear epitopes (Luo et al., 2020, 2021). The immunogenicity of these novel antigens was tested against HME human convalescent sera to show that they were recognized after infection (Luo et al., 2020, 2021). Although this study identified novel antigens none of them were tested as vaccine candidates, so it remains to be determined if any or a combination of them are protective.

Another recent study used subtractive genomics and reverse vaccinology to identify surface exposed proteins with linear B cell epitopes (Sabzi et al., 2023). A multi-epitope vaccine was designed and used in a C-ImmSim immunoreactivity simulation to demonstrate a strong Th1 response, however, this would also have to be demonstrated in animal models (Sabzi et al., 2023). The influx of genome sequences for different *E. chaffeensis* strains will help in identifying antigens using subtractive genetics and reverse vaccinology to develop a safe and efficacious vaccine.

One of the other major challenges for vaccine development for *Ehrlichia* spp. is the lack of appropriate animal models. The outer membrane protein P28 (OMP-19) has a long history of protection studies in different animal models of *E. chaffeensis*. The earliest study demonstrated that intraperitoneal immunization of P28 could prevent blood-borne infection of *E. chaffeensis* in BALB/c mice, which was a feat that the time but is not a physiologically relevant animal model. After this initial study, passive transfer studies showed that monoclonal antibodies to P28 OMP could prevent disease caused by *E. chaffeensis* in SCID mice (Shu-Yi Li et al., 2001). This study had to be done in SCID mice since *E. chaffeensis* does not infect wild-type mice in a physiologically relevant manner. Therefore, many of the other vaccine studies used *E. muris* as a surrogate in mice, for mild virulence in humans, or IOE in mice, for fatal infection in humans, to test the protective ability of P28 (Nandi et al., 2007; Crocquet-Valdes et al., 2011).

The study with IOE established that P28-19 could provide partial protection as not all immunized mice survived with high humoral and cell-mediated responses indicating a Th1 skew (Nandi et al., 2007). Vaccination and challenge with P28 and *E. muris* demonstrated IFN-γ specific CD4+ T cells and increased IgG2c after vaccination and challenge indicating a Th1 skewed response and again provided partial protection (Crocquet-Valdes et al., 2011). Another study using vaccination with peptide epitopes from P28 and Hsp60 and challenge with *E. muris* demonstrated similar results which consisted of partial protection and a Th1 skew (Thomas et al., 2011).

Additionally, a TRP protein P29 from *E. muris* has been used in vaccination challenge experiments to show a reduction in bacteria in the liver and spleen but not the lung and blood in a high dose challenge (Thirumalapura et al., 2013). All these studies demonstrate that there are immunogenic proteins that induce a Th1 skewed response but none of these provide protection alone.

The development of transposon mutagenesis in *E. chaffeensis* in 2013 led to the testing of several attenuated mutants as vaccine candidates (Cheng et al., 2013). Two mutants with *in vivo* growth defects were tested as live attenuated vaccine candidates in dogs (incidental host) and deer (reservoir host) with promising results (Nair et al., 2015). These results were extended by demonstrating that vaccination with Ech_0660 transposon mutant provided significant protection followed by challenge in dogs after tick-transmission of *E. chaffeensis* and induced both humoral, Th1, and Th17 responses (Mcgill et al., 2016). After this first report of protection, vaccination with entry trigger protein Etp-E also demonstrated accelerated clearance from dogs as was observed using the attenuated live vaccine (Budachetri et al., 2020). This study was followed up with another study using the same tick-transmission model and two other subunit vaccines OMP-1B and VirB2-4 which caused clearance of *E. chaffeensis* at an earlier time-point after tick transmission (Budachetri et al., 2022).

All these studies demonstrate that there are very good immunogenic proteins in *E. chaffeensis* and the right combination of them with a Th1 skewing adjuvant could provide protection. Also, very interestingly, the induction of Th17 responses with the live attenuated vaccine is important as these responses are important in the clearance of other intracellular bacteria.

## *Anaplasma* spp. vaccines

As with *E. chaffeensis*, the major barrier to the development of a vaccine against *A. phagocytophilum* is antigen choice. Although a lot of research has been done to develop a vaccine against *A. marginale* in cattle, very little progress has been made to accomplish this goal against *A. phagocytophilum* for humans. Once again, *A. phagocytophilum* lacks LPS, and therefore glycoconjugate vaccine strategies are irrelevant. MSP2(P44) is an immunodominant OMP of *A. phagocytophilum* which was determined using human convalescent serum. However, only the N and C termini are conserved (Jw et al., 1997). The central hypervariable region (HVR) is varied using pseudogenes and a gene combinatorial conversion mechanism (Barbet et al., 2003). Therefore, although antibody responses to MSP2(P44) show some level of protection, the extreme variation of this protein makes it a poor candidate for a universal vaccine (Kim and Rikihisa, 1998).

On the other hand, *A. phagocytophilum* encodes 3 surface-exposed adhesins, which could serve as targets for an antibody-mediated vaccine to prevent cell invasion. Antibodies targeting domains that prevent binding of OmpA, Asp14, or AipA reduced infection in host cells *in vitro* by 50%, whereas, using a combination of all 3 reduced infection by 80% (Seidman et al., 2015). This laid the groundwork to determine the relevance of these 3 adhesins to infection *in vivo* by immunizing mice with keyhole limpet conjugated peptides from the binding domains of each (Naimi Waheeda et al., 2020). Interestingly, only antibodies to the binding domains of Asp14 and AipA inhibited *in vitro* infection after vaccination (Naimi Waheeda et al., 2020). This vaccine strategy also induced antigen-specific IFNγ-producing CD8+ T cells but not CD4+ T cells (Naimi Waheeda et al., 2020). IFNγ is important for the control of *A. phagocytophilum* presumably by activating macrophages. Although it was only partially protective, the addition of other T cell antigens or the use of a different adjuvant like CpG to induce a better Th1 response could elicit better protection.

Another strategy for an *A. phagocytophilum* vaccine is to use subdominant protein antigens from the type IV secretion system VirB9 and VirB10 a strategy that has been more widely investigated for *A. marginales* (Crosby et al., 2018). The protection by VirB10 was marginal at best but did produce antigen-specific IFNγ+ CD4+ T cells suggesting that a vaccine strategy that includes VirB10 with other antigens in combination with an appropriate adjuvant may provide a better level of protection (Crosby et al., 2018).

## Tick vaccines

A strategy that has emerged for the treatment of tick-borne diseases is to vaccinate against the tick instead of the pathogen carried by the tick. The acquired resistance or so-called “tick immunity” after repeated tick bites was first described by Trager in 1939 and is associated with inflammation at the site of tick bite (Trager, 1939). This type of resistance usually develops in non-natural hosts (Narasimhan et al., 2021). Understanding how this resistance develops has helped the development of tick-specific vaccines. In the most general terms, anti-tick vaccines target both concealed and non-concealed protein antigens which are exposed after the tick starts a blood meal. Strategies include targeting tick attachment, feeding, and salivary gland proteins (De La Fuente et al., 2020). The BM86-based vaccine for control of cattle tick infestations is the only licensed tick vaccine marketed as Gavac™ in Cuba and TickGUARD^PLUSTM^ in Australia (Willadsen et al., 1989). As resistance has grown to chemical acaricides, this strategy has become the lead candidate to combat ticks and disease transmission in livestock. The development of anti-tick vaccines has also benefited from machine learning and reverse vaccinology. An *A. phagocytophlium* specific vaccinomic approach used previously published transcriptomic and proteomic data on *A. phagocytophilum* infected *I. scapularis* to identify candidate protective antigens and determine their homologs in the closely related *I. ricinus* (Contreras et al., 2017). This strategy identified 2 potential tick-protective antigens that are involved in pathogen infection and transmission.

More recently, a cocktail of 19 salivary proteins based on a previous analysis of the *Ixodes scapularis* sialome was used to create a nucleoside-modified mRNA-encapsulated lipid nanoparticle for vaccinated guinea pigs (Sajid et al., 2021). None of the vaccinated guinea pigs were positive for *Borrelia burgdorferi* while 46% of the unvaccinated guinea pigs were culture-positive for *B. burgdorferi* (Sajid et al., 2021). Erythema at the site of tick attachment was a hallmark of tick-resistance (Sajid et al., 2021). This strategy should also work for *A. phagocytophilum* which is transmitted by *I. scapularis* and other ixodid ticks. The most attractive part of anti-tick vaccines is that they should prevent any transmission of infectious agents carried by the said tick.

## Discussion

This review provides a broad overview of what is known about the immune responses to Rickettsiaceae, *Coxiella burnetti*, and *Anaplasmataceae* and the challenges that have been faced in developing safe and efficacious vaccines to date. The identification and testing of a handful of antigens for each of the obligate intracellular bacteria has taken at least half a century. It is our view that any vaccination strategy that stimulates a Th1 mediated response with the production of pathogen specific antibodies, CD4+ T cell, and CD8+ cytotoxic T cell will be most effective for any of the obligate intracellular bacteria as discussed throughout this review. The next phases of novel vaccine development for this group of obligate intracellular pathogens will use the new vaccinology approaches described in this review based on genomics and proteomics, with the inclusion of machine learning algorithms to define protective antigens. Once these antigens or epitopes are identified we believe their delivery using the licensed mRNA and viral vectors DNA used during the SARS-CoV-2 pandemic should provide optimal protection because they induce the optimal protection described above providing both CD4+ T and CD8+ T cells responses in addition humoral responses. Additionally, these delivery methods do not rely on producing subunit antigens which has hampered the development of these vaccines as they are often difficult to produce. These innovations should allow for the development and testing of safe and effective vaccines soon for *Rickettsiaceae*, *C. burnetti*, and *Anaplasmataceae*. The challenges that remain will be the development of CoPs that can be used to define protection by CD4+ and CD8+ T cells in the clinical phases of testing. Although defining CoPs remains a challenge the other innovations discussed in this review should greatly excelerate the identification and testing of novel antigens. The need for these vaccines will continue as the rise of infections and expansion of vector territories for these obligate intracellular bacteria continues.

## Author contributions

EV: Conceptualization, Writing – original draft, Writing – review & editing. AF: Writing – original draft, Writing – review & editing. AG: Writing – original draft, Writing – review & editing. JD: Writing – original draft, Writing – review & editing. JS: Funding acquisition, Writing – original draft, Writing – review & editing.
